# Improving Indicators in a Brazilian Hospital Through Quality-Improvement Programs Based on STS Database Reports

**DOI:** 10.5935/1678-9741.20150075

**Published:** 2015

**Authors:** Pedro Gabriel Melo de Barros e Silva, Antônio Claudio do Amaral Baruzzi, Denise Louzada Ramos, Mariana Yumi Okada, José Carlos Teixeira Garcia, Fernanda de Andrade Cardoso, Marcelo Jamus Rodrigues, Valter Furlan

**Affiliations:** 1 Amil Chronic Diseases Management Unit (Totalcor), São Paulo, SP, Brazil - Brazilian Clinical Research Institute (BCRI), São Paulo, SP, Brazil; 1 Amil Chronic Diseases Management Unit (Totalcor), São Paulo, SP, Brazil

**Keywords:** Coronary Artery Bypass, Quality Improvement, Database Management Systems

## Abstract

**OBJECTIVE:**

To report the initial changes after quality-improvement programs based on STS-database in a Brazilian hospital.

**METHODS:**

Since 2011 a Brazilian hospital has joined STS-Database and in 2012 multifaceted actions based on STS reports were implemented aiming reductions in the time of mechanical ventilation and in the intensive care stay and also improvements in evidence-based perioperative therapies among patients who underwent coronary artery bypass graft surgeries.

**RESULTS:**

All the 947 patients submitted to coronary artery bypass graft surgeries from July 2011 to June 2014 were analyzed and there was an improvement in all the three target endpoints after the implementation of the quality-improvement program but the reduction in time on mechanical ventilation was not statistically significant after adjusting for prognostic characteristics.

**CONCLUSION:**

The initial experience with STS registry in a Brazilian hospital was associated with improvement in most of targeted quality-indicators.

**Table:** 

**Abbreviations, acronyms & symbols**	
CABG	= Coronary artery bypass grafting
ICU	= Intensive care unit
LOS	= Length of stay
MV	= Mechanical ventilation
OR	= Odds Ratio
QI	= Quality improvement
STS	= Society of Thoracic Surgeons

## INTRODUCTION

Large scale analysis has shown that the adherence to evidence-based quality indicators is associated with better clinical outcomes^[1]^. Standardized quality control programs are useful to improve these indicators and have shown progressive importance in cardiovascular surgery^[2]^. Society of Thoracic Surgeons (STS) Database is a strong source of information for quality programs, considering that this registry includes data from more than one thousand hospitals in a wide range of indicators.

The STS Database separates the hospitals according to their performance in diverse indicators delivering benchmarks for their participants. This tool has shown utility for the development and evaluation of quality improvement (QI) programs^[3,4]^. However, most of this evidence comes from studies in North America and little is known in other regions. The value of this STS registry may not be applicable in other countries outside of the United States of America. Since July 2011, a Brazilian hospital has joined the STS Database. The current report aims to show the first three years of experience and the initial local changes on quality-indicators through QI programs based on STS database reports.

## METHODS

### Population analyzed

All consecutive patients submitted to isolated coronary artery bypass graft surgeries (CABG) from July 2011 until June 2014 in a Brazilian private cardiovascular hospital were analyzed.

### Intervention

A multifaceted and continuous educational program based on STS reports was implemented during the year of 2012 by a multidisciplinary team targeting three indicators: 1) Time on mechanical ventilation (MV); 2) Length of stay (LOS) in intensive care unit (ICU); 3) Evidence-based perioperative therapies in eligible patients including the use of internal mammary artery in CABG. A local team composed by physicians and nurses developed an institutional protocol for the appropriate use of perioperative medications (including sedation during perioperative period) and implemented a systematic assessment to the first 6 hours after surgery using objectives criteria for extubation. The QI team trained physicians, nurses and physiotherapists to follow the institutional protocol and also used the data from the best hospitals in STS report as benchmark to show to the surgical and clinical staff that the targets were feasible. A case manager nurse was responsible to prospectively collect the data from each patient, enter the data in the institutional database and STS database, and also to check the indicators, making interventions with the team in case of non-adherence to hospital goals.

### Choice of target endpoint

The hospital has joined STS Database in July 2011 and the first reports were provided in the end of 2011 and beginning of 2012. The three targeted endpoints described above in the intervention section have been chosen through multidisciplinary meetings including clinicians, surgeons, nurses, intensive care staff, anesthesiologist and physiotherapists identified local priorities according to the team interpretation of these reports. Reintubation and readmission to ICU were also monitored to evaluate safety of early extubation and discharge.

### Secondary endpoints

STS Database quarterly reports each Hospital's performance in the domains of morbidity, mortality and overall composite (Table 1) rating each hospital from one to three stars according to the differences between the hospital's performance compared to the average results of the other hospitals included in this database.

**Table 1:** Domains of the STS Database report.

Domain	Description
Avoidance of operative mortality	Proportion of patients (risk-adjusted) who do not experience operative mortality
	Operative mortality is defined as death during the same hospitalization as surgery or after discharge but within 30 days of the procedure
Avoidance of major morbidity	Major morbidity is defined as having at least one of the following adverse outcomes: 1) reoperations for any reason; 2) renal failure; 3) deep sternal wound infection; 4) prolonged ventilation/ intubation; 5) cerebrovascular accident/permanent stroke
Proportion of patients (risk-adjusted) who do not experience any major morbidity	
Use of internal mammary artery (IMA)	Proportion of first-time CABG patients who receive at least one IMA graft without prior CABG surgery or documented contraindication for IMA use (subclavian stenosis, previous cardiac or thoracic surgery, previous mediastinal radiation, an emergent salvage procedure or no LAD disease)
Use of all evidence-based perioperative medications	Proportion of patients who receive all required perioperative medications: 1) preoperative beta blockade therapy; 2) discharge anti-platelet medication; 3) discharge beta blockade therapy; and 4) discharge anti-lipid medication. Note: the discharge medications were not required for patients who died prior to discharge
Overall composite	A participant's overall composite performance score is calculated as a weighted* average of the domain-specific estimates

* The weights that appear in the composite score equation were chosen based on an empirical analysis in which the standard deviation of each of the four endpoints was estimated. These weights are updated following each harvest since they are based upon the specific analysis of each harvest's data. This is the example of the equation used during the period analyzed: CAB Composite Score = 0.71xscoremort + 0.10xscoremorb + 0.15xscoreIMA + 0.04xscoremeds.

### Comparison

Two groups were compared in order to observe changes after the QI programs: those who underwent surgery before (July 2011 until December 2012) *vs.* after the complete implementation of the multifaceted program (January 2013 until June 2014).

### Statistical analysis

For primary endpoints, a maximum limit of significance of 5% was defined for chance of type I error (*P*<0.05) in two-tailed tests. Continuous variables were presented as means and standard deviation for cases that proximity could be determined with a normal distribution. The Student's *t* test was used to compare means. The Wilcoxon and Mann-Whitney tests were applied to other continuous variables; presented by means and interquartile ranges (25^th^ and 75^th^ percentile). Data related with categorical variables were analyzed using the chi-squared test or the Fisher exact test when necessary. The two target endpoints that may be influenced by prognostic factors (time on MV and LOS in ICU) were also included in a logistic regression multivariate analysis to evaluate the independent association of these indicators with the period of the program (2011/2012 *vs.* 2013/2014). The following covariates were included in this analysis: age, gender, hematocrit, creatinine (pre and post), cross-clamp and perfusion times, comorbidities, STS risk. Statistical calculations were done using the Statistical Package for Social Sciences program (SPSS, Chicago, IL, USA) version 20.0.

For analysis of quality domains in CABG procedures, statistical significance is based on a 99% Bayesian certainty criterion. The Bayesian credible interval shows the range in which the participant's true proportion (or risk-adjusted proportion) is likely to lie. A participant receives 3 stars if there is at least 99% Bayesian probability that the participant's score exceeds the STS mean score. A participant receives one star if there is at least 99% Bayesian probability that the participant's score is less than the STS mean score. Otherwise, the participant receives 2 stars. The rates for the mortality and morbidity domain are adjusted to the risk of the patients in each hospital to avoid confounders.

The current analysis followed the ethical aspects described in the Declaration of Helsinki and the Document of the Americas. Our study was approved by the Institutional Ethical Review Board.

## RESULTS

From July 2011 to June 2014, 947 CABGs were performed. Comparing the pre and post-QI program population, they had similar mean age (61.8 *vs.* 60.3 *P*=0.07) and percentage of elective procedures (54.3% *vs*. 49.8% *P*=0.17) but more females submitted to CABG pre-QI (26.4% *vs.* 16.8% *P*<0.01). Despite baseline differences in specific items such as gender, a validated multivariate model (STS score), showed that the predicted risk of In-hospital mortality during the period analyzed was 0.75% in 2011, 1.1% in 2012, 0.75% in 2013 and 0.7% in 2014 (*P*>0.05). As exposed in Table 2, there was a reduction in the time on MV, in the postoperative LOS and also improvement in the use evidence-based perioperative medications. The improvement in the use of internal mammary artery was not statistically significant. The rates of reintubation and readmission were not different in both groups (Table 2). Using a risk-adjusted model (STS score), the reduction in LOS and in the time on MV were associated with more patients discharged with less than 6 days but the difference of prolonged MV (> 24 hours) was not statistically significant (Table 2). A multivariate analysis showed that controlling for prognostic factors the chance of staying > 48 hours in ICU was higher in 2011/2012 when compared to 2013/2014 (OR=1.45; CI 95% 1.06-1.86; *P*=0.017). When analyzed the chance of staying > 24 hours on MV, this indicator was also higher in 2011/2012 when compared to 2013/2014 but the odds ratio (OR) was not statistically significant in a multivariate analysis (OR=1.28; CI 95% 0.89-1.70; *P*=0.12).

**Table 2:** Results of the quality-improvements targets.

	Pre-QI program (n=519)	Post- QI program (n=428)	*P*-value
Time on MV (hours)	10.4	7.1	< 0.01
Initial ventilation < 6 hours	52%	85%	< 0.01
Reintubation	2.9%	2.1%	0.53
O/E MV > 24 hours (95% CI)	0.45 (0.24 - 0.66)	0.24 (0.04 - 0.45)	ns
LOS in ICU	64.9	54.2	< 0.01
Readmission to ICU	4.4%	2.8%	0.22
O/E LOS < 6 days (95% CI)	0.80 (0.70-0.90)	1.03 (0.92 - 1.14)	0.02
Use of IMA	98.2%	99%	0.40
Use of EBT	87.4%	95.3%	< 0.01
Avoidance of operative mortality	98.50%	98.60%	0.99
Avoidance of major morbidity	91.10%	95.10%	0.02
O/E mortality ratio (95% CI)	1.5 (0.97 - 2.03)	1.7 (1.0- 2.4)	ns

QI=quality improvement; MV=mechanical ventilation; LOS=length of stay; ICU=intensive care unit; IMA=internal mammary artery; EBT=evidence-based therapy; O/E=observed/expected; ns=non-significant

The overall composite domain (see definition in Table 1) was rated as 2 stars in 2012 and improved to 3 stars in 2013. The specific domains were rated as 2 stars during the period pre-intervention, improving to three stars in 2013 only the domains of medication and absence of morbidity (absence of mortality and use of IMA still rated as two stars and also post-educational intervention).

## DISCUSSION

The first three years of experience showed that the use of a large international registry as STS Database as a benchmark to guide QI programs in a Brazilian Hospital was associated with improvement in target quality-indicators. There was a reduction in all the three endpoints targeted. Reintubation and readmission did not increase with an earlier extubation and discharge.

The main goals for the years of 2013 and 2014 were defined by the local group after evaluation of the first STS Database reports. Considering that time on MV, LOS in ICU and use of appropriate therapy cannot be modified by a single intervention, a multifaceted program was necessary to achieve reduction in these indicators.

The use of the own hospital database for QI is a common practice in many services, including Brazilian Hospitals^[2]^ but the use of multicenter registries for continuous QI programs as STS Database had not been available until recently in Brazil despite their use for years in such countries as the United States^[1-4]^. This tool for evaluation of medical performance was developed to assess relevant evidence-based indicators adjusted to the risk of the patients. The innovation of the present report is that despite cultural barriers and demographic diversity among patients and professionals, this tool showed a very promising application in a new country. Similar to the majority of QI programs implemented in clinical practice, the multifaceted QI program reported was not developed primarily for research purposes but aimed mainly improvement in local indicators along the time in a real world scenario. The difference of the reported QI program is that it was based on quarterly reports of an international database including a Brazilian hospital.

The comparison with different hospitals was extremely useful to identify the most important discrepancies that need to be addressed. However, analysis of outcomes measurements (e.g. mortality, cerebrovascular accident) in this kind of registry should be evaluated carefully because differences in these results may be related to the risk of the patient and not the quality of care. Even considering that STS reports provide the results adjusted to the risks, we made a local validation of these models^[5]^ to be more confident when comparing the results of the hospitals. The current report did not target mortality endpoint which, in our point of view, probably will need more time, more patients and more QI programs to change along the time.

The only targeted item that did not show a statistically significant improvement after the QI program was the use of internal mammary artery. This finding is probably related to the fact that the baseline performance was very close to the target and it would need a large number of patients to show significant difference. Despite the absolute 8% improvement in the use of evidence-based medications, a gap of 5% still remained. This gap is almost entirely related to preoperative beta blockade therapy, which remain an area of debate regarding the benefit of its routine use in the preoperative period of CABG^[6]^. Thereby, not only the educational intervention but also the strength of the evidence is important to change behavior and improve quality indicators.

The lack of a randomized control group makes the current analysis of endpoints vulnerable to confusion factors. Multivariate analysis was performed to evaluate if the period after the QI program was associated with improvement in the target indicators independently of other prognosis covariates. In addition, prognosis differences are more important in comparisons of clinical outcomes, which was not the main objective of this analysis that targeted indicators related to the process of care. Regarding the use of evidence-based therapy in eligible patients this indicator is not influenced by prognostic factors and multivariate analysis was not considered necessary, especially because the groups compared had similar prognosis characteristics along the years using STS model for in-hospital mortality. Also, these patients were treated in the same service and by the same medical team, and this report intends to represent a real-world utilization of the STS Database.

Other aspect to be considered is that the interventions were specific to three indicators and that this is a single center experience. This concept should be evaluated in more hospitals and different clinical scenarios to confirm the applicability of this tool for a broader use in Brazilian cardiovascular centers.

## CONCLUSION

The initial experience with STS registry in a Brazilian hospital was associated with improvement in most of targeted quality-indicators.

**Table:** 

**Authors' roles & responsibilities**	
PGMBS	Analysis/interpretation of data; statistical analysis; final approval of the manuscript; conception and design; implementation of projects/experiments; manuscript writing or critical review of its content
ACAB	Analysis/interpretation of data; final approval of the manuscript; manuscript writing or critical review of its content
DLR	Analysis/interpretation of data; final approval of the manuscript
MYO	Analysis/interpretation of data; final approval of the manuscript
JCTG	Analysis/interpretation of data; final approval of the manuscript
FAC	Analysis/interpretation of data; final approval of the manuscript; implementation of projects/experiments
MJR	Analysis/interpretation of data; final approval of the manuscript; implementation of projects/experiments
VF	Analysis/interpretation of data; final approval of the manuscript; conception and design; manuscript writing or critical review of its content
